# 238. Comparison of Outcomes of Patients with Gram-Negative Bacterial Left Ventricular Assist Device-Related Infections on Chronic Suppressive Antibiotic Therapy versus Patients Monitored on No Antibiotic Therapy

**DOI:** 10.1093/ofid/ofad500.311

**Published:** 2023-11-27

**Authors:** Petros Svoronos, Nicholas Marschalk, Sajed Sarwar, Brent Lampert, Courtney Nichols

**Affiliations:** The Ohio State University Wexner Medical Center, Columbus, Ohio; The Ohio State University Medical Center, Columbus, Ohio; The Ohio State University Wexner Medical Center, Columbus, Ohio; Ohio State Wexner Medical Center, Columbus, Ohio; OSU Wexner Medical Center, Columbus, Ohio

## Abstract

**Background:**

Left ventricular assist devices (LVAD) are used for short- and long-term support in heart failure. LVAD-related infections may manifest as infections of the driveline or bacteremia and may result in morbidity and mortality. Treatment may involve surgical debridement and prolonged antimicrobial therapy. Given concern for recurrent infection due to retained hardware, suppressive antibiotics are commonly utilized after the index infection. However, minimal data is available to guide these decisions.

**Methods:**

This was a single-center, retrospective cohort study at our institution from November 1, 2011 to September 1, 2021 comparing outcomes after index LVAD-related gram negative driveline infections or bacteremia who received suppressive antibiotic therapy versus those who did not. Patients on suppressive antibiotics for previous gram-positive infections were excluded. Fischer’s exact test was used to evaluate for statistically significant differences between the two groups.

**Results:**

Of 460 patients who underwent LVAD placement, 32 patients met inclusion criteria. Demographic data and infection related outcomes are summarized in Tables 1 and 2. Number of infection recurrence (61.5% vs 57.9%) and LVAD-related infection re-admissions (69.2% vs 73.7%) were similar between the two groups. A higher proportion of patients started on chronic suppressive therapy during the index infection had surgical management (53.8% vs 36.8%) and bacteremia as their index infection (30.8% vs 18.8%). Subsequent cultures positive for multidrug resistant (MDR) organisms (15.4% vs 10.5%) and infection-related mortality (15.4% vs 0%) were also both observed to be higher in the group on chronic suppressive antibiotics. No statistically significant difference was observed in outcomes between the two groups (Table 2).

**Figure:**
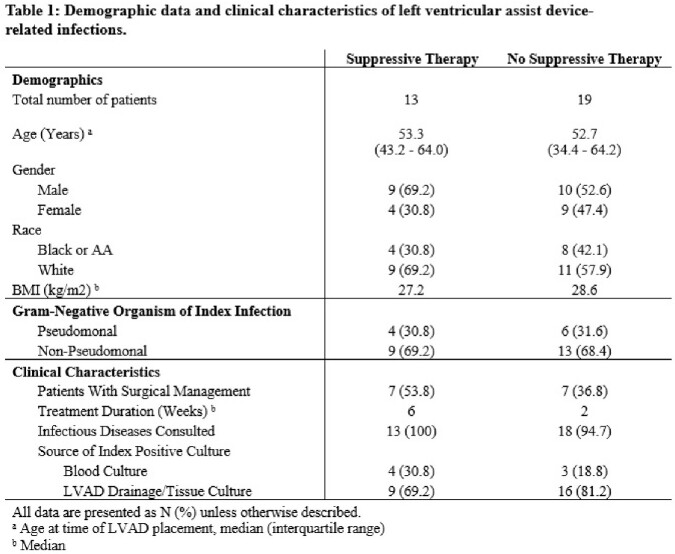

**Figure:**
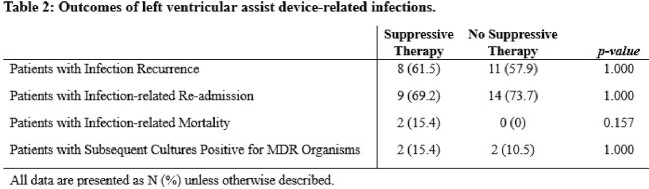

**Conclusion:**

Following index gram-negative LVAD-related infections, patients transitioned to suppressive antibiotic therapy were observed to have similar outcomes compared to those monitored off chronic antibiotic therapy at our institution. Future studies with a larger sample size are necessary to determine if there is a statistically significant benefit to suppressive antibiotic therapy after gram-negative LVAD-related infections.

**Disclosures:**

**All Authors**: No reported disclosures

